# Supplementary material for: A Humanized Yeast Phenomic Model of Deoxycytidine Kinase to Predict Genetic Buffering of Nucleoside Analog Cytotoxicity
Source: Genes (Basel). 2019 Sep 30;10(10):770. doi: 10.3390/genes10100770 (PMC6826991; doi:10.3390/genes10100770)
Supplement: Supplementary file 1 [file genes-10-00770-s001.zip › Additional_Files/Additional_File7_GOTermHeatmaps.zip!/Additional_File7_GOTermHeatmaps/mitotic_DNA_replication_checkpoint.pdf]

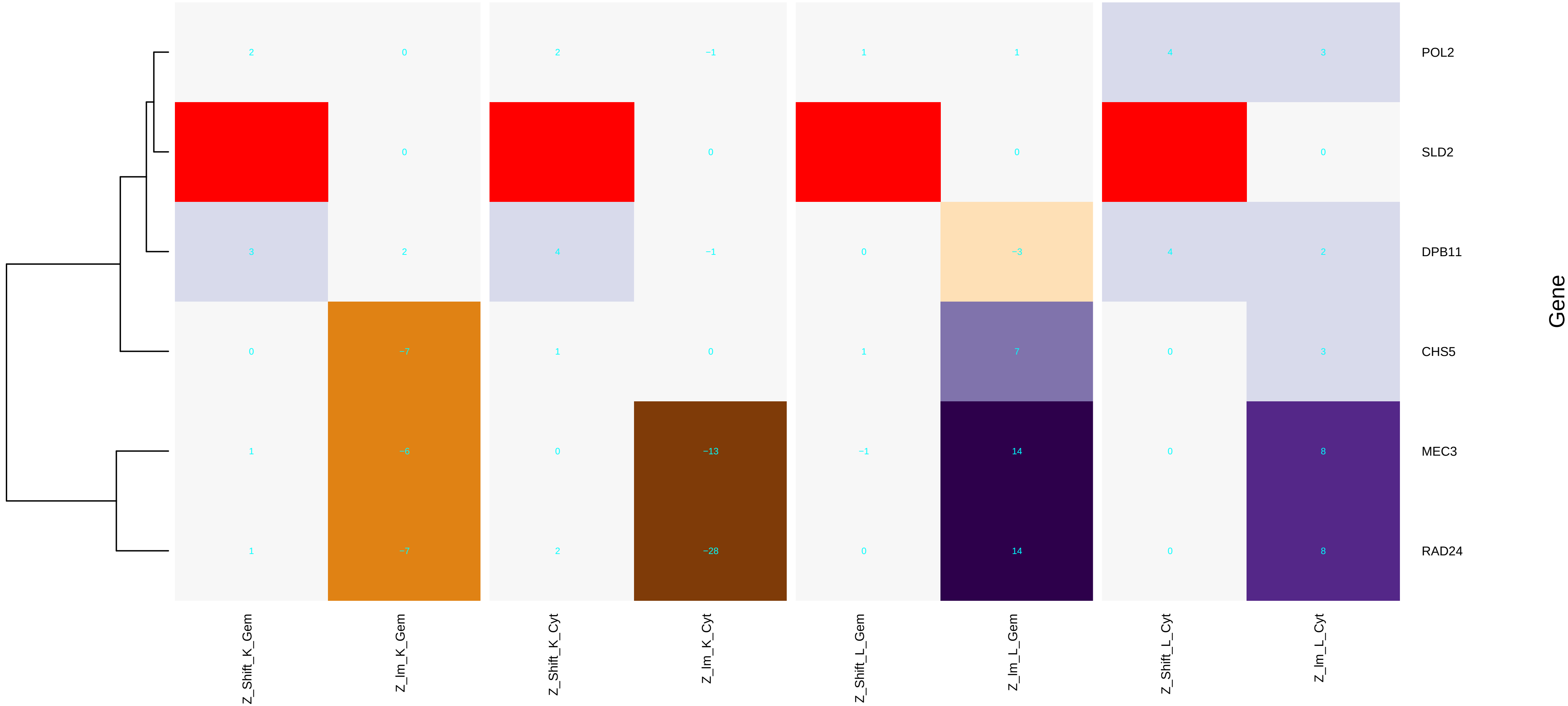
POL2
SLD2
DPB11
CHS5
MEC3
RAD24
Gene
Z_Shift_K_Gem
Z_Im_K_Gem
Z_Shift_K_Cyt
Z_Im_K_Cyt
Z_Shift_L_Gem
Z_Im_L_Gem
Z_Shift_L_Cyt
Z_Im_L_Cyt
2 0 2 −1 1 1 4 3
0 0 0 0
3 2 4 −1 0 −3 4 2
0 −7 1 0 1 7 0 3
1 −6 0 −13 −1 14 0 8
1 −7 2 −28 0 14 0 8
